# A Variable-Length Chromosome Genetic Algorithm for Time-Based Sensor Network Schedule Optimization

**DOI:** 10.3390/s21123990

**Published:** 2021-06-09

**Authors:** Van-Phuong Ha, Trung-Kien Dao, Ngoc-Yen Pham, Minh-Hoang Le

**Affiliations:** 1 MICA Institute (HUST—Grenoble INP), Hanoi University of Science and Technology, Hanoi 100000, Vietnam; van-phuong.ha@mica.edu.vn (V.-P.H.); ngoc-yen.pham@mica.edu.vn (N.-Y.P.); minh-hoang.le@mica.edu.vn (M.-H.L.); 2 Faculty of Electrical Engineering Technology, Hanoi University of Industry, Hanoi 100000, Vietnam

**Keywords:** wireless sensor networks, variable-length chromosome genetic algorithms, schedule optimization, planning optimization, energy efficiency

## Abstract

Scheduling sensor nodes has an important role in real monitoring applications using sensor networks, lowering the power consumption and maximizing the network lifetime, while maintaining the satisfaction to application requirements. Nevertheless, this problem is usually very complex and not easily resolved by analytical methods. In a different manner, genetic algorithms (GAs) are heuristic search strategies that help to find the exact or approximate global optimal solution efficiently with a stochastic approach. Genetic algorithms are advantageous for their robustness to discrete and noisy objective functions, as they are only evaluated at independent points without requirements of continuity or differentiability. However, as explained in this paper, a time-based sensor network schedule cannot be represented by a chromosome with fixed length that is required in traditional genetic algorithms. Therefore, an extended genetic algorithm is introduced with variable-length chromosome (VLC) along with mutation and crossover operations in order to address this problem. Simulation results show that, with help of carefully defined fitness functions, the proposed scheme is able to evolve the individuals in the population effectively and consistently from generation to generation towards optimal ones, and the obtained network schedules are better optimized in comparison with the result of algorithms employing a fixed-length chromosome.

## 1. Introduction

Wireless sensor networks have been particularly appealing in research in many fields, such as environmental monitoring, healthcare, industrial production inspection, agriculture, energy, transportation, security, military, as well as in civil applications [1]. Thanks to the advantages such as flexibility, customizability, and easy deployment in large-scale and complex environments, wireless sensor networks have been becoming more and more widely used.

Optimization has an important role in the research, development, and application of sensor networks, where the problems are related to energy consumption, network coverage, communication routing, network lifetime, etc. The optimization objectives are also very diverse. For example, in maximizing network lifetime, the criteria can be defined differently, either the network is considered to exist when only a few nodes remain active or when its perceptible range is above a certain threshold [2]. Moreover, in reality, a network can be heterogeneous in terms of sensor type, the number of nodes is large, and the deployment environment is broad and complicated. Therefore, sensor network optimization problems are generally complex as they usually involve multiple subjects.

Among network optimization problems, schedule optimization is commonly used in deploying sensor networks with goal to reduce the energy consumption, increase the network lifetime, while guaranteeing its activities to satisfy the constraints specified by applications. Basically, this is achieved based on timely programming the working modes in each node individually so that they cooperate and accomplish intended tasks. However, while all strategies have a common design objective to maximize network lifetime, the mechanisms to solve this problem are as diverse as the techniques used in sensor network deployment due to different assumptions considered in the context of different applications [3]. Therefore, the mechanisms can be categorized in many ways: static and dynamic, centralized and distributed, communication-based and communication-less cooperation, hierarchical and non-hierarchical, etc.

As an example of communication-based cooperation, Miller and Vaidya in [4] developed a special MAC-layer power saving scheme using a wake-up radio to minimize the transceivers’ idle time, hence reduce the communication power consumption in a non-hierarchical network. A two-radio architecture is used so that a sensor is able to wake-up a neighbor with a trigger and send its packets to that destination. In another study [5], Nawaz et al. developed a cooperative scheduling mechanism with physical-layer communication scheme to avoid collisions of multiple data flows in large-scale networks using orthogonal frequency-division multiplexing (OFDM) to reduce the energy consumption. In [6], Nguyen et al. applied sleeping schedules for sensor nodes together with cognitive radio techniques to reduce frequency band usage, and compressed sensing to reduce data transmission, hence cut off energy consumption in the network. The cooperative approach is generally more flexible and responses more adaptively in complex applications when external factors change quickly and working context is highly unpredictable. However, in typical application classes, such as surveillance monitoring, where these conditions are more deterministic, the transmission overhead for cooperation messages would unnecessarily consume energy and shorten the nodes’ lifetime.

Therefore, whenever possible, communication-less cooperative planning would be more optimal in terms of energy effectiveness. In principle, this can be fulfilled with the help of strategies that are based on information coming from ambient factors [7,8] where time is one of the mostly used for its availability and the simplicity in implementation. In this study, a time-based schedule optimization problem is considered, where sensor nodes are of active-then-sleep type. As time schedule is discrete by nature, searching for the optimal solution using analytical methods is unsuitable. We aim to approach this problem using genetic algorithms (GAs) [9] as a leading generic searching mechanism for global optimal solution in complex search spaces. A GA uses a string called chromosome to encode the parameters, which would correspond to the network schedule in this case. Due to the method formulation, especially in supporting the genetic operations, the chromosome structure is fixed, i.e., the number of elements as well as their order are unchanged over all individuals. However, in principle, at least there is no limit on the number of state changes that are performed by each sensor node. In other words, the network schedule is flexible and cannot fit into classical GAs whose chromosomes are fixed in length and are not able to represent the schedule information. Besides GAs, many other evolutionary and swarm-intelligence optimization algorithms have been proposed in the literature, among them the mostly discussed ones include ant colony optimization (ACO) [10], particle swarm optimization (PSO) [11], fruit fly optimization algorithm (FFOA) [12], firefly algorithm [13], bacterial foraging algorithm (BFA) [14], or more recently, monarch butterfly optimization (MBO) [15], earthworm optimization algorithm (EWA) [16], elephant herding optimization (EHO) [17], moth search (MS) algorithm [18], slime mould algorithm (SMA) [19], Harris hawks optimization (HHO) [20], grasshopper optimization algorithm (GOA) [21], battle royale optimization algorithm (BROA) [22], etc.

From the basic form, many techniques have been introduced to improve these algorithms, mostly the performance of the algorithm in terms of convergence speed. As a recent example, Wang et al. [23] proposed an information feedback mechanism that considers not only the current individuals in the reproduction phase, but also those from the last one, two, or three generations. This technique can be applied to any metaheuristic optimization algorithm, and its superiority has been proven for many of those, including ACO, PSO, and MBO. However, all these algorithms are based on a common property that the optimization parameters are encoded by a vector which characterizes the individuals, while this vector is not only fixed in length but also its structure. With this parameter vector, the fitness function and reproduction mechanism can be developed. This study does not aim to improve the convergence performance for none of these algorithms, but to broaden the representation space of the parameter vector by loosening its constraints in order to extend the applicability of these algorithms. The introduced technique is illustrated with GA as the most well-known and basic nature-inspired metaheuristic optimization algorithm, so that the principle is better explained, but in principle can also be adopted to any other one, as it is independent from the algorithm itself.

In this study, a modified GA with variable-length chromosome (VLC) extension is developed to address this problem. The most challenging issue to formulate a VLC-GA is to adapt the genetic operations, and to evaluate the fitness function which is now a programmatical rather than a mathematical one. A number of variations of VLC-GAs have been introduced in the literature for several applications. In [24], the authors proposed a VLC to represent QoS-aware multi-path web-service composition plans so that the optimal path can be determined using a GA. To do this, the customer service requirements are modeled using a directed graph, then the service selection and composition problem is transformed into a pattern matching problem of service travel flow. The crossover operation is implemented based on cut and splice operations on the graph paths, whereas the mutation operation is just like other GAs. Very closed to this approach, the authors in [25] introduced a VLC-GA for a system that solves planning problems, i.e., finding the best sequence of actions to achieve given goals. Also in this graph-based paradigm, Cruz-Piris et al. [26] developed a VLC-GA to solve a road traffic coordination multi-path problem, in which the chromosome is broken down into cells that encode path branches. In terms of complex chromosome, genetic programming (GP) [27] is an analogous evolutionary mechanism to search for optimal computer programs, but is limited to problem that can be represented in a tree-based structure.

Regarding sensor networks, a large number of studies have been using GAs in different ways for optimization problems [28], but only few research attempts adopted VLC-GAs. In [29], Deif and Gadallah proposed a VLC-GA to solve a wireless sensor network deployment problem whose objectives are maximizing coverage and minimizing the deployment cost. In their study, the chromosome is composed of a variable number of integers that correspond to labels of deployment tuples in a pre-calculated set, where a tuple consists of three parameters, including sensor type, deployment point, and orientation angle. Another study is introduced in [30], which employed a VLC-GA in the path design of a mobile sink going through fixed nodes in a wireless sensor network to collect data. The design objective is to balance the global network energy consumption and hence maximizing the network lifetime. The chromosome in that study is a list of integers representing the node identifiers.

It can be observed that most of the aforementioned studies as well as others in the literature that adopt VLC-GA are graph based, where a chromosome represents a path in the graph, and genes are of integer type. Furthermore, in those studies, although the chromosome length is variable, but is limited to a specific range, hence the number of available combinations is also limited. In contrast, the VLC-GA proposed in this study makes use of real-typed genes, and the chromosome has no limit in length.

The remainder of this paper is organized as follows. In Section 2, the schedule optimization problem for sensor networks is introduced. After that, classical GAs are briefly reviewed in Section 3, whereas the proposed VLC-GA to solve the network schedule problem is formulated and explained in Section 4. In Section 5, some case studies with simulation results are given and discussed. Finally, concluding and perspective remarks are given in Section 6.

## 2. Sensor Network Schedule Optimization Problem

This section discusses the schedule optimization problem of sensor networks. In a wide range of monitoring applications, sensors need to be deployed in environments that lack power supply conditions or present mobility requirements. In addition, even in indoor environments, small ubiquitous sensors have nowadays been freed from communication cables by dozens of wireless transmission technologies such as ZigBee, LoRa, Wi-Fi, Bluetooth, cellular, etc., as power cables become an obstacle for their application in reality. For these reasons, the energy supply and consumption of sensor networks in general need to be carefully optimized.

There are several available approaches for self-powered wireless sensors. Instrumenting nodes with battery is among the first options due to the simplicity in implementation as well as the usability. However, this is obviously not a radical solution for applications that require long-term activity or are deployed in human-inaccessible fields. Wireless power transfer is an emerging solution but so far, the achieved effective range is still far from being suitable for real applications [31,32]. Researchers have been also seeking to develop nodes that harvest ambient energy by converting from available sources in surrounding environment such as solar, wind, heat, vibration, radiofrequency (RF) radiation, etc. However, most ambient energy sources cannot guarantee to provide regularly and sufficiently to relax users from power constraints. Therefore, in any case, lowering power consumption is essential in order to ensure the longevity of sensor networks. This leads to many studies for the optimization of the nodes’ working schedule, besides efforts seeking to use low-power electronic components.

**Figure 1:**
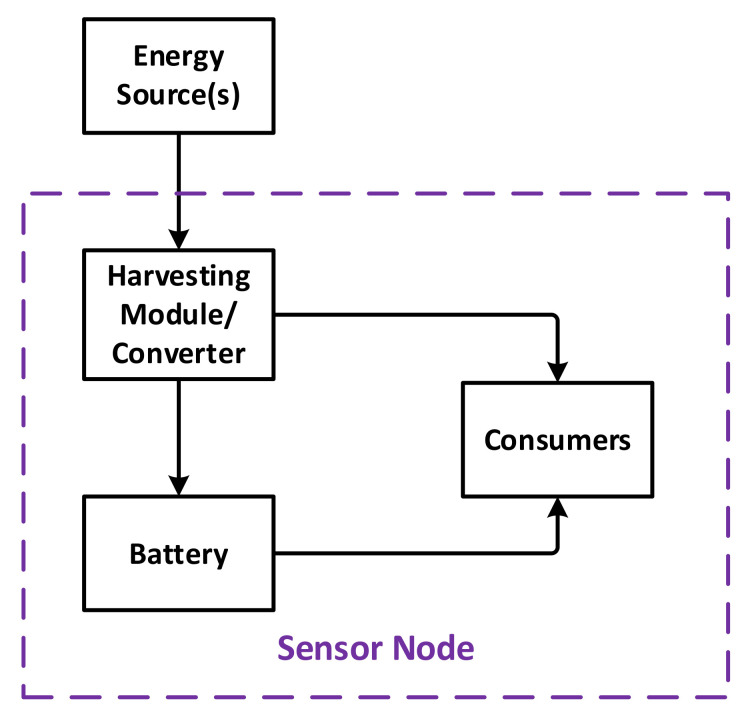
Flow of energy in a typical sensor node.

In this study, a general model of sensor nodes is used, as shown in Figure 1. Regarding the energy, a node consists of three major components: a power unit, a storage unit, and consumers. The consumers include every element in the node that consumes energy, such as communication, processing and sensing elements. The power unit can be a harvesting module that helps the node to collect ambient energy, or to convert the energy from other external sources [33,34]. Finally, the battery unit is used to store energy when not utilized directly. On the design basis, a consumer element may drain energy from the power unit or the battery, or both. It is also necessary to note that one of the power units or the battery may be omitted, and when the power unit is present, the battery is usually rechargeable.

Suppose that the network to be scheduled is composed of *n* nodes indexed from 1 to *n*, which may be, but are not necessarily identical. Each node, based on its own deployment conditions, may collect ambient energy at certain rate and time period. The goal is seeking to schedule each node to maximizing a specific goal of achievement provided by this network, while ensuring the energy-related constraints.

For a typical monitoring application, each sensor node in a particular time may function in one of two modes, namely active and sleep, each of them is characterized by a different average rate and pattern of power consumption. In active mode, the node is able to execute its programmed tasks such as sensing, measurement, data processing, data receival and transmission, etc., whereas in sleep mode, only a small low-power part of the node functions and carries out minimal activities to maintain the readiness for future activation. In more general cases, the nodes may have more than two modes. Denote the set of possible modes for node i as $M^{i}$, then a schedule $S^{i}$ for node i is specified by a sequence of pairs:

(1) $$S^{i}≜{\left.\langle \left(m_{j}^{i},t_{j}^{i}\right)\rangle \right|}_{j=1\ldots s^{i}}^{},$$

where $s^{i}$ is the length of this sequence, i.e., the number states for node i; $m_{j}^{i}\in M^{i}$ is the mode used in state j; and $t_{j}^{i}$ is the starting moment of this state. In this study, for simplicity, only active-then-sleep sensors are considered, i.e., $M^{i}=\left\{\bar{M},\underline{M}\right\}$ for every node, where $\bar{M}$ is the active mode that corresponds to a higher power consumption level, whereas $\underline{M}$ is the sleep mode. Note that, although the nodes have the same modes, they may have different power consumption characteristics. Moreover, the power consumption rates are not constant, but are assumed to be predictable.

A schedule $\hat{S}$ of the whole network is just the combination of the schedules for every node, i.e.,

(2) $$\hat{S}={\left.\left\{S^{i}\right\}\right|}_{i=1\ldots n}^{}.$$

Eventually, $t_{1}^{i}=0$ and $t_{s^{i}}^{i}=T$ for every node, where T is the end time of the schedule.

With above definitions, the problem to be solved now is to find the optimal schedule of the whole network that maximizes or minimizes a predefined objective function, and guarantees a set of constraints. The objective function and constraints need to be chosen carefully to accurately reflex the superiority of a given schedule, and take into account the rules that restrict the system’s activity, respectively. As an example of constraints, sensor nodes are usually not able to switch between modes arbitrarily, but need to follow a mechanism that is specified by design. Regarding the sensor network deployment, the most-frequently used objective functions are usually related to energy consumption, lifetime, measurement coverage, and communication characteristics.

Theoretically, there is no limit for the number of states of one sensor node. This makes the schedule search space become very large. Therefore, the dependency of the objective function on the schedule is highly non-trivial and not suitable to be modelled or solved by traditional methods such as analytical ones. Preferable alternative methods are usually related to heuristics, such as machine-learning-based or evolutionary ones.

In this study, we aim to propose a technique find the solution to this problem using a genetic algorithm with variable-length chromosome. To use genetic algorithms, one needs to define the chromosome structure that encodes the problem input, that is the network schedule in this case.

## 3. Overview of Genetic Algorithms

A genetic algorithm is an optimization problem solving method that uses a heuristic approach. In optimization problems, the most important thing is the objective function, which can be generally represented by a multi-variable mathematic function that maps elements from some input domain X to real numbers:

(3) $$f\left(x\right):X\to \mathbb{R},$$

where x∈X is the variable vector. Normally, X is a subset of elements in ${\mathbb{R}}^{n}$ that satisfy the constraints. As for minimization problem, a solution $x_{0}$ is an element that $f\left(x_{0}\right)\leq f\left(x\right)$ for all x∈X.

In genetic algorithms, a chromosome C is an ordered list of genes and corresponds to an element x in the input domain X. However, as it is used in a computer algorithm, then to facilitate the presentation in programming languages, it is usually given in the form of an array of values that describes the input element by encoding its characteristics. For this reason, in a classical genetic algorithm, the chromosome length is fixed, and is n in most cases. Regarding the terminology, in genetic algorithms, input elements are called individuals, and the objective function is usually referred as fitness function as it helps to score the supremacy of the individuals.

**Figure 2:**
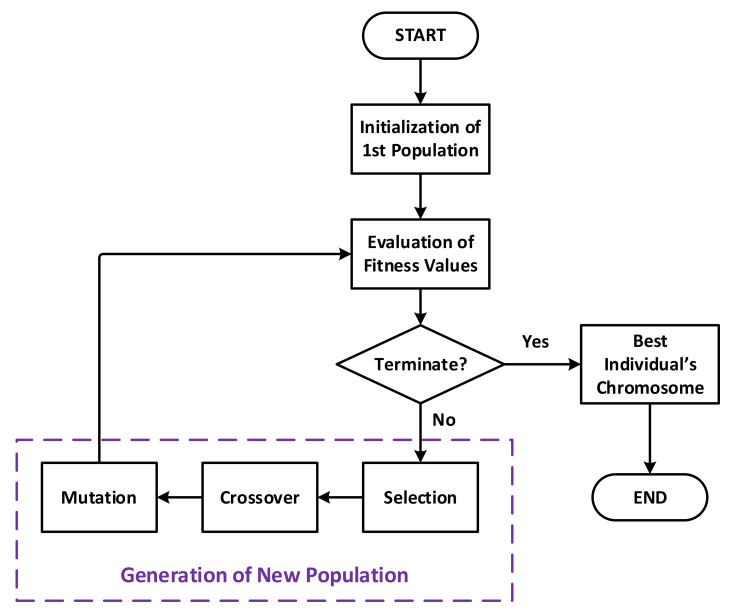
Flowchart of a genetic algorithm.

In its most basic form, the execution of a typical genetic algorithm can be represented by the flowchart depicted in Figure 2. For the first generation, when the algorithm starts, the individuals may be randomly generated, or with help of one or a few seeding individuals provided by the user as an algorithm input.

The principal execution process is a main loop that helps to gradually evolve the population generation by generation, each one corresponds to one loop iteration. The loop only terminates when the quality of the population is good enough, i.e., accepted by a given termination condition, which is usually related to the fitness values. As the optimal fitness value is unknown in theory, one cannot know its absolute threshold value a priori. Therefore, the termination condition is very often defined by using its derivatives, such as the difference of the average fitness value over generations, for example.

For a population generation, the loop iteration starts by evaluating the fitness value of every individual. If the termination condition is not satisfied, the process will continue by creating new children that make up the next generation in such a manner that its quality is improved in comparison to the parent one. This step is also referred as reproduction, and is achieved by performing three genetic operations called selection, crossover, and mutation on the current population:

- Selection keeps the best individuals in the population based on their fitness values. The selected individuals survive and remain as they are to the next generation.
- Crossover takes pairs of parent individuals and randomly combines their genes to create children.
- Mutation takes a parent chromosome and introduces random changes to make children.

Note that the order of these operations may differ, or they may even be combined in different ways in some variations of the algorithm to have more flexibility in the implementation, e.g., a child individual may suffer from all these operations. There are also different strategies of choosing parent individuals for these operations.

For selection, a popular implementation is using a certain percentage $\rho_{S}$ of elite individuals to be kept, but one may use a probability distribution function (PDF) $f_{S}\left(i\right)$ that favor the elite individuals and disfavor the inferior ones. In the same manner, similarly PDFs, denoted as $f_{C}\left(i\right)$ and $f_{M}\left(i\right)$, can be applied when choosing input parents to the crossover and mutation operations, respectively.

## 4. Variable-Length Chromosome Genetic Algorithm for Network Scheduling Problem

In this section, the solution of the network scheduling problem using a proposed VLC-GA is introduced. The first subsection is the theoretical formulation and implementation techniques of the algorithm, and the later one briefly introduces a platform used for energy-aware simulation of sensor networks.

### 4.1. Formulation of Variable-Length Chromosome Genetic Algorithm

#### 4.1.1. Chromosome Structure

For the network scheduling problem, the chromosome C needs to encode the network schedule $\hat{S}$. In this study, it is defined as

(4) $$\begin{array}{r} C=\left[s^{1},m_{1}^{1},t_{1}^{1},m_{2}^{1},t_{2}^{1},\ldots ,m_{s^{1}}^{1},t_{s^{1}}^{1},\right.\\ s^{2},m_{1}^{2},t_{1}^{2},m_{2}^{2},t_{2}^{2},\ldots ,m_{s^{2}}^{2},t_{s^{2}}^{2},\\ \ldots ,\\ \left.s^{n},m_{1}^{n},t_{1}^{n},m_{2}^{n},t_{2}^{n},\ldots ,m_{s^{n}}^{n},t_{s^{n}}^{n}\right]. \end{array}$$

The number of genes for node i is $\left(2s^{i}+1\right)$, and for the whole network is $\left(2\sum_{i=1}^{n}s^{i}+n\right)$. As $s^{i}$ are variable, the length of C is also variable. It is important to note that in a VLC-GA, the fitness function is not a mathematical function in common sense, but rather a programmatical function, with takes the chromosome as input and returns the fitness value as output, after an execution process. In contrast to fixed-length chromosomes where the genes are located at fixed location, in a VLC, they are not. For this reason, $s^{i}$ are included in C so that computer programs can interpret the genes correctly, otherwise it is not possible to know where the schedule information for each node starts and ends.

Basically, as C varies from individual to individual (remind that an individual corresponds to a network schedule configuration) and from generation to generation, then to express its dependency on the individual, denote ${}^{q}C\left(k\right)$ the chromosome of individual q in generation k. Similarly, all other individual-dependent variables also follow this notation convention as well, e.g., ${}^{q}m_{j}^{i}\left(k\right)$ is state j of node i in individual q in generation k.

Also, denote ${}^{q}C^{i}$ the segment in ${}^{q}C$ that correspond to the schedule of node i, that is,

(5) $${}^{q}C^{i}=\left[{}^{q}m_{1}^{i},{}^{q}t_{1}^{i},{}^{q}m_{2}^{i},{}^{q}t_{2}^{i},\right.\left.\ldots ,{}^{q}m_{{}^{q}s^{i}}^{i},{}^{q}t_{{}^{q}s^{i}}^{i}\right].$$

Note that the length of ${}^{q}C^{i}$ is $2{}^{q}s^{i}$, and ${}^{q}C$ can now be simplified as

(6) $${}^{q}C=\left[{}^{q}s^{1},{}^{q}C^{1},{}^{q}s^{2},{}^{q}C^{2},\right.\left.\ldots ,{}^{q}s^{n},{}^{q}C^{n}\right].$$

**Figure 3:**
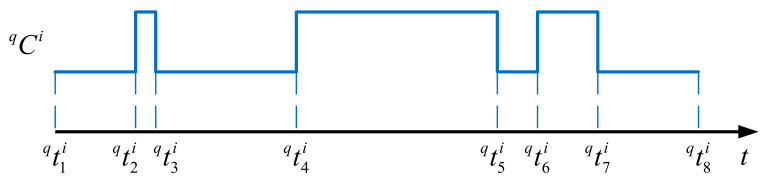
Graphical representation of a node schedule.

For visualization, ${}^{q}C^{i}$ can be illustrated graphically as shown in Figure 3. The mode switching events made by node i are presented along the time axis, where the higher-level segments correspond to the periods when the node is active, whereas the lower ones represent the periods when the node is in sleep mode.

In order to implement a VLC-GA, it is necessary now to adapt the reproduction operations. The detailed implementation is explained in the remaining of this section. Obviously, the Selection operation does not need any modification for adaptation, only the two other operations do.

#### 4.1.2. Crossover Operation

Regarding the crossover operation, since in principle, each sensor node in a network may perform a different role, mating two different nodes has very little interest but would severely increase the algorithm complexity. Therefore, in this study, the mating is performed on the node basis. More clearly, the crossover operation is applied to the chromosomes from the same node in the two individuals, but not others. In other applications, one can easily go further with cross-node mating as extension, if particularly interested. At this point, the crossover operation on individuals is broken down to that on corresponding nodes.

**Figure 4:**
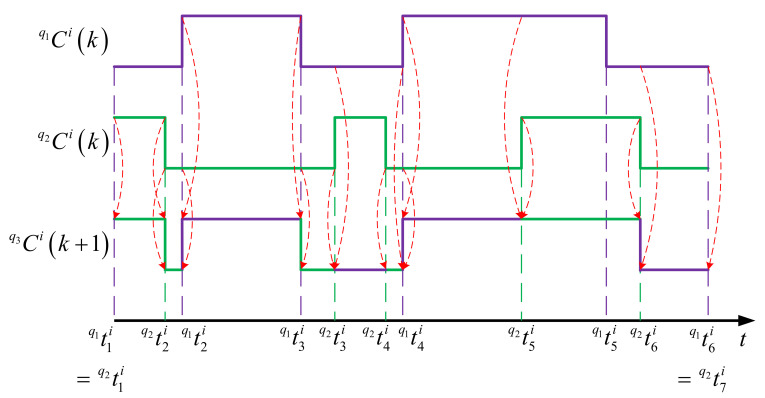
Crossover operation on sensor node schedule.

The principle of the crossover operation is illustrated by the example shown in Figure 4, where ${}^{q_{1}}C^{i}\left(k\right)$ and ${}^{q_{2}}C^{i}\left(k\right)$ are the parent chromosomes of a same node i but from two different individuals $q_{1}$ and $q_{2}$ in generation k, and ${}^{q_{3}}C^{i}\left(k+1\right)$ is the resulting child chromosome of the same node in the next generation $\left(k+1\right)$.

To perform this operation, the intervals from the two mating nodes’ schedules are first sorted in increasing order of timestamp with redundant ones excluded, so that the running time $\left[0,T\right]$ can be broken down into intervals. For the above example, the resulting time intervals are $\left[{}^{q_{1}}t_{1}^{i},{}^{q_{2}}t_{2}^{i}\right]$, $\left[{}^{q_{2}}t_{2}^{i},{}^{q_{1}}t_{2}^{i}\right]$, $\left[{}^{q_{1}}t_{2}^{i},{}^{q_{1}}t_{3}^{i}\right]$, $\left[{}^{q_{1}}t_{3}^{i},{}^{q_{2}}t_{3}^{i}\right]$, etc. After that, for each interval, the mode for the child node is determined by randomly picking a mode at the same interval from one of the two parents. In the figure, purple intervals in ${}^{q_{3}}C^{i}\left(k+1\right)$ represent the those inherited from ${}^{q_{1}}C^{i}\left(k\right)$, whereas green ones correspond to the those inherited from ${}^{q_{2}}C^{i}\left(k\right)$. To favor the genes from elite parents, higher probability can be associated to the modes from the parent with higher fitness value in the mode selection, and vice versa.

Finally, in the resulting chromosome, if two or more consecutive intervals have a same mode, they are combined for simplification. That is, $\left[{}^{q_{1}}t_{3}^{i},{}^{q_{2}}t_{3}^{i}\right]$, $\left[{}^{q_{2}}t_{3}^{i},{}^{q_{2}}t_{4}^{i}\right]$, and $\left[{}^{q_{2}}t_{4}^{i},{}^{q_{1}}t_{4}^{i}\right]$ in the example are all in sleep mode and are simplified to $\left[{}^{q_{1}}t_{3}^{i},{}^{q_{1}}t_{4}^{i}\right]$.

#### 4.1.3. Mutation Operation

**Figure 5:**
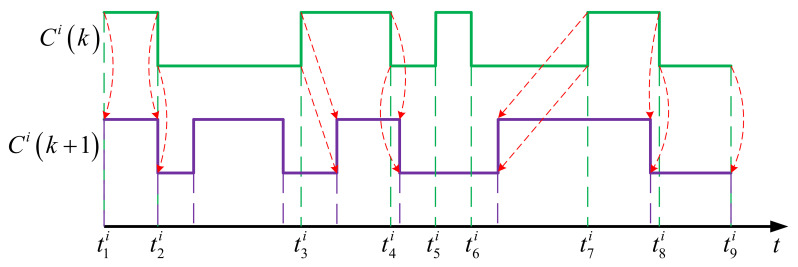
Mutation operation on sensor node schedule.

For Mutation operation, the principle is illustrated by the example shown in Figure 5. Starting from the parent chromosome $C^{i}\left(k\right)$, for each interval, one randomly chosen of the following four operations is carried out to produce the new one $C^{i}\left(k+1\right)$:

- Copy: The child interval is same as the parent interval, like the intervals $\left[t_{1}^{i},t_{2}^{i}\right]$ and $\left[t_{8}^{i},t_{9}^{i}\right]$ in the example.
- Insertion: A new interval with inverse mode is inserted in a manner that its start and end times are generated randomly but stay within the boundaries of the parent one, as in the case of the interval $\left[t_{2}^{i},t_{3}^{i}\right]$ in the example.
- Removal: The parent interval is removed and not inherited by the child node, as in the case of the interval $\left[t_{5}^{i},t_{6}^{i}\right]$ in the example.
- Shift: The boundaries of the child interval is made by moving those of the parent one backward or forward in the time axis, as in the case of the intervals $\left[t_{3}^{i},t_{4}^{i}\right]$ and $\left[t_{7}^{i},t_{8}^{i}\right]$ in the example.

These operations are performed at given rates $\rho_{M}^{C}$, $\rho_{M}^{I}$, $\rho_{M}^{R}$, $\rho_{M}^{S}$, corresponding to copy, insertion, removal, shift, respectively, where $\rho_{M}^{C}+\rho_{M}^{I}+\rho_{M}^{R}+\rho_{M}^{S}=1$.

For the example shown in the figure, the first interval $\left[t_{1}^{i},t_{2}^{i}\right]$ is affected by a copy operation, the second one $\left[t_{2}^{i},t_{3}^{i}\right]$ is affected by an Insertion operation with a new subinterval randomly inserted in the middle, the third one $\left[t_{3}^{i},t_{4}^{i}\right]$ is affected by a shift operation with boundaries randomly moved rightward, the fifth one $\left[t_{5}^{i},t_{6}^{i}\right]$ is affected by a removal operation and disappears from the schedule.

The above mechanism of selection, crossover and mutation helps the algorithm to explore any possibility of network schedule by diversifying the generation of genes as the individuals evolve, and at the same time prioritize the good genes that make the corresponding individuals superior. Finally, it is necessary to mention that $s^{i}$ elements in C are not subjected to the crossover and mutation operations, but are updated as a result of the application of these operations on corresponding $C^{i}$ elements in C.

### 4.2. Energy-Aware Simulation of Sensor Networks

For any optimization method, one obligatory requirement is that, for any variable value x∈X, it is possible to evaluate the objective function $f\left(x\right)$. Regarding our problem with sensor networks, in principle, to evaluate the fitness function, it is necessary to run the network in demanded conditions and measure the suitable parameters. However, heuristic methods like GAs usually need to evaluate the fitness function a huge number of times, hence the above method of fitness evaluation is just not realistic. To address this problem, a simulator is essential.

**Figure 6:**
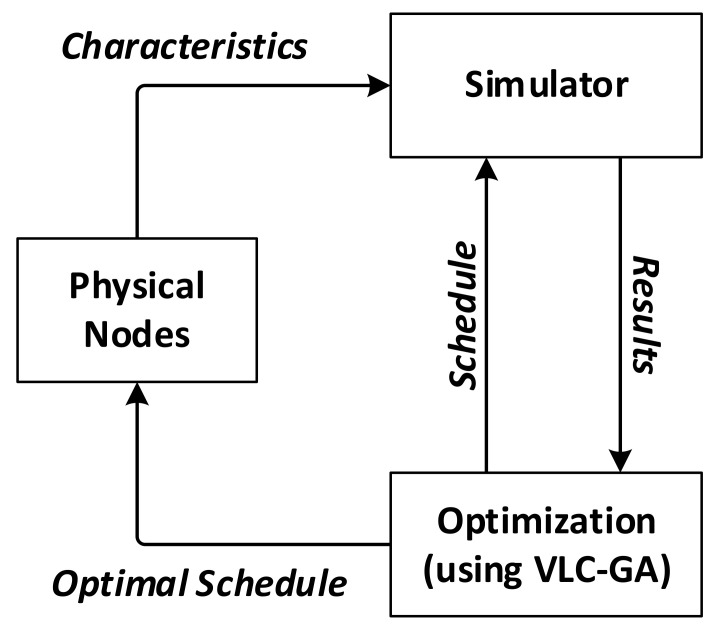
Schedule optimization workflow.

The workflow for schedule optimization is depicted in Figure 6. For given physical sensor nodes, their functioning mechanism as well as power-consumption characteristics are replicated by simulated nodes in the simulation platform. As stated earlier in this paper, the power consumption pattern in each mode of the sensor nodes is assumed to be predictable. Once the simulator is established, the proposed VLC-GA would be able to call it for any schedule and obtain back the required results for fitness evaluation. On termination of the optimization algorithm, the resulting optimal schedule is transferred and implemented into the physical nodes. The simulation platform is briefly introduced in the remaining part of this subsection.

**Figure 7:**
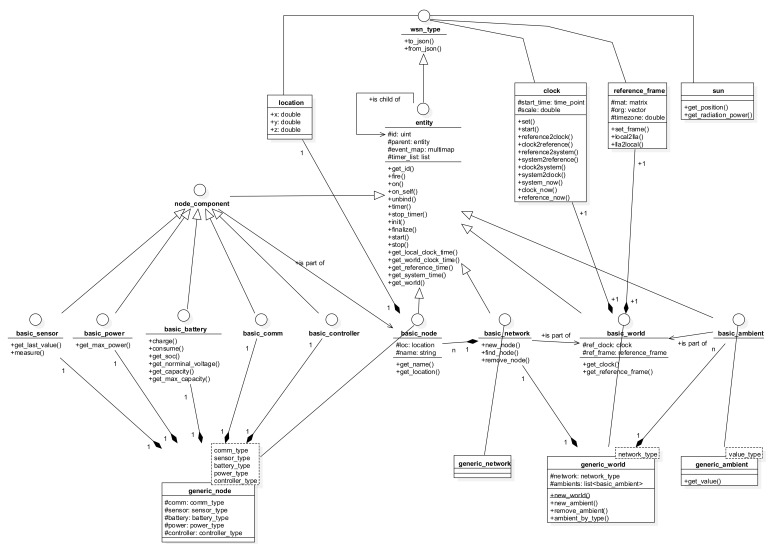
Class diagram of the simulation platform.

The simulation platform is developed and implemented in C++ using highly modulated generic template classes designed with event-driven approach, in order to accomplish both execution performance and programming flexibility, with the general class model shown in Figure 7. Besides auxiliary classes that implement the location, coordinate frames, time, and clocks, entity is the base interface that represents any “living” entities such as networks, sensor nodes, and sensor components. A living entity is characterized by a working thread dedicated to its functionalities, including the event propagation and capturing mechanism.

Derived from entity, the principal interface and class groups include:

- Node components, whose base interface is node_component. A node component can be of one among five types, i.e., battery, power, sensor, communication, and controller, which are explained below.
- Sensor nodes, whose base interface is basic_node.
- Networks, whose base interface is basic_network.
- Working environments, whose base interface is basic_world.
- Ambient environmental elements, with basic_ambient. An ambient environmental element represents an external physical field, such as temperature, light, humidity, etc., that has impact on the functioning of the sensor network. Examples of possible impacts are energy source for the power components, or measurement values for the sensor components.

**Figure 8:**
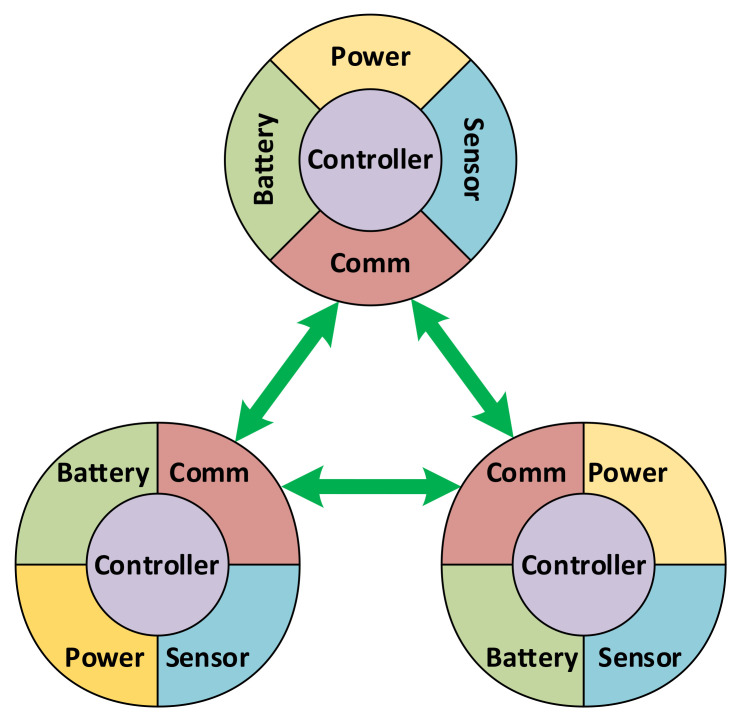
Design of sensor node components in simulation platform.

Basically, a simulation is set up with a world which consists of a network and a number of necessary ambient elements. A network includes individual user-defined nodes. Each node in turn is a combination of five components that are implemented individually, of five component types, as shown in Figure 8:

- Battery: used to simulate the energy storage behaviors. A library of common battery types, including lead acid, Li-ion, Ni-Cd and Ni-MH, is implemented as part of this platform, using the models given in [35].
- Power: responsible for collecting power from external sources, including ambient energy harvesting as well as wired or wireless sources feeding. Regarding the simulations in this work, a solar energy source is used to charge the batteries using the model given in [36].
- Sensor: used to simulate the sensing mechanism.
- Communication: implementing low- and high-level communication protocols. Note that the communication is for data routing required by other purposes, not for schedule cooperation, as earlier stated in this paper.
- Controller: implementing the functioning and incorporation of the four other components in the node.

**Figure 9:**
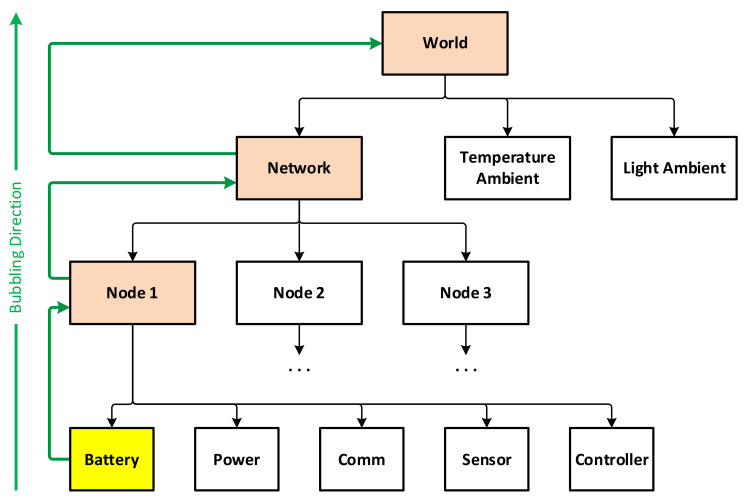
How the event bubbling works in the hierarchy.

Events in this simulation platform are provided according to a propagation mechanism with bubbling ability. An event that is triggered at an entity is also successively propagated to the entities at higher levels that own it, allowing events to be registered and captured not only by the same entity, but also by its parent and ancestors. Consider the example in Figure 9, a network has multiple sensor nodes, and each node has its components. When an event is triggered by a component of a certain node, it is possible to register a handler for this event right there. However, if the same event is triggered by multiple components of a node, it would be more convenient to capture that event at the node level to simplify the handler. Similarly, in order to capture that event for the components of different nodes in the network, it is also possible to capture at the network level. The bubbling process can also be canceled at any level by a handler.

A multi-thread executor is implemented so that each entity, such as network, sensor node, or sensor component, is executed in a separate thread. This helps to make them independent and not blocking each other, while are still able to communicate with each other freely. Furthermore, ambient physical phenomena such as meteorological, geospatial are also implemented and available for usage. This is important when, for example, nodes consume solar energy and this needs to be predicted accurately at a specific time and at a specific location on Earth. For further reference, this simulation platform has been introduced in more details in [37] and its source code is available at: https://github.com/daotrungkien/wsnsim (accessed 8 June 2021).

**Figure 10:**
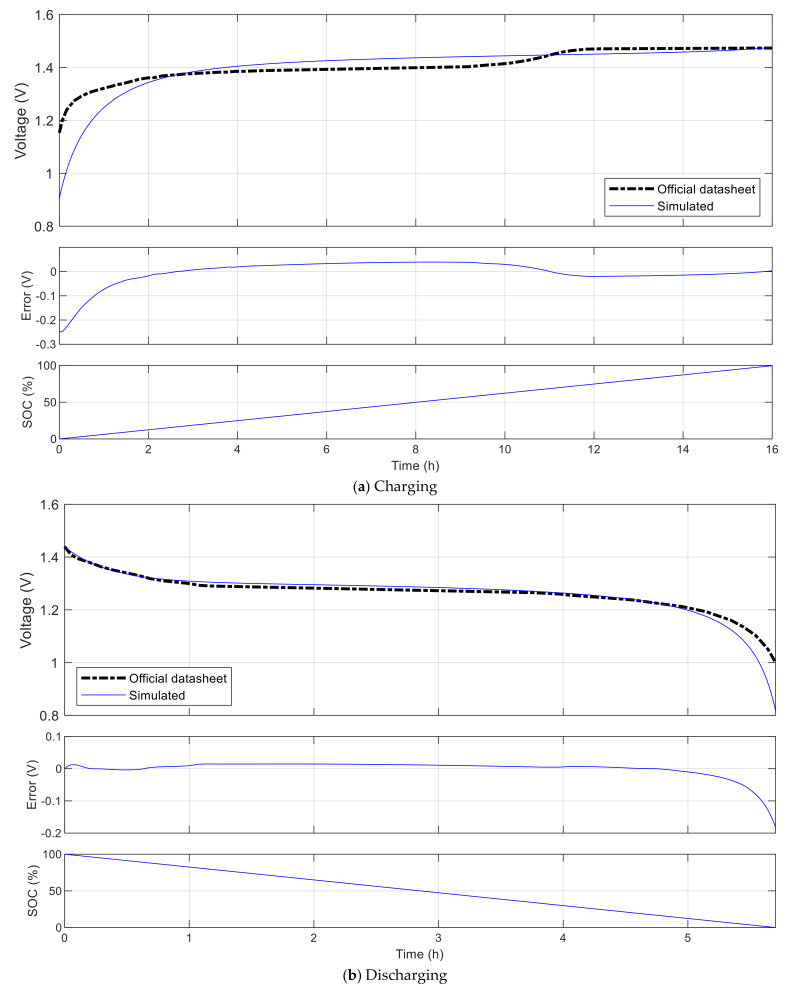
Validation simulation of the Panasonic BK-60AAAH battery.

Figure 10 shows validation results when comparing the charge and discharge characteristics of the Panasonic BK-60AAAH battery simulated with the platform and the reference behavior published by the producer in their officially datasheet [38]. This is a Ni-MH battery whose principal characteristics include nominal voltage of 1.2 V, cutoff voltage of 1 V, capacity of 550 mAh. In the first simulation, the battery is charged from empty state to full using a constant charging current of 50 mA, then the state of charge (SOC) increases gradually from 0% up to 100% in 16.0 h, and the voltage increases to the fully charged voltage of 1.47 V with the root mean square error (RMSE) of 0.049 V. In the second simulation, the battery is discharged from full state to empty using a constant current of 100 mA, then the SOC decreases gradually from 100% down to 0% in 5.7 h, and the voltage decreases from the fully charged voltage with the RMSE of 0.026 V. In both cases, the highest errors occur when the battery is in the total discharge zone, i.e., the SOC is too low and the voltage increases or decreases rapidly. If we exclude this zone and consider only the nominal-to-full zone, which is normally considered as the working zone of a battery, then the voltage RMSE for charging and discharging are 0.025 V and 0.001 V, respectively.

**Figure 11:**
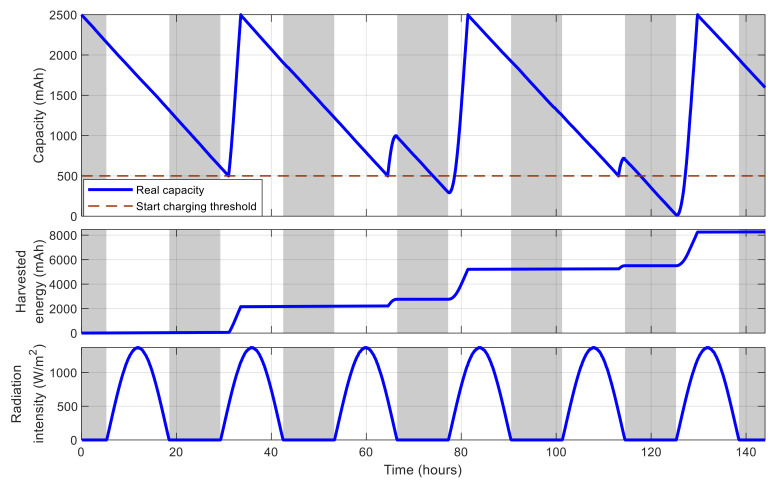
Energy-related behavior of an example simulated sensor node.

To demonstrate the simulation platform, Figure 11 shows the battery capacity and the cumulated harvested solar energy of an example simulated sensor node equipped with a rechargeable battery of 2500 mAh in 144 h. The node’s location is assumed to be 21.004° in latitude and 105.846° in longitude. In this figure, the gray strips express the nighttime, i.e., the time from sunset until sunrise, whereas the white ones correspond to daytime. The node is programmed so that it consumes energy from its battery at a constant rate for its internal usage, but only starts charging when the battery level is lower than a predefined threshold of 20%, and stops charging when the battery is fully charged. It can be observed that when the node is not charging, the battery capacity degrades gradually until encountering the threshold, and the node starts charging. However, as the solar energy is only available in the daytime, there are moments in nighttime when the node is charging but the battery level still degrades because there is no sunlight. Moreover, from the figure, it can also be observed that the charging rate is limited in the early morning and late afternoon periods, as the solar radiation intensity is low.

## 5. Case Studies and Simulation Results

In this section, two scenarios designed to demonstrate the proposed VLC-GA are introduced.

### 5.1. Single-Node Case

In this scenario, a network with only one node is considered to monitor certain environmental parameters within a period of three days with a daily schedule, i.e., the schedule for each day remains unchanged. The node is designed with two modes, namely active and sleep, and consumes solar energy collected by a panel and is disposed with a battery to store redundant power. The solar radiation rate is variable of location on Earth and time in a manner that it is high in daytime and low in nighttime. It can be deduced based on the real solar position which is calculated with the algorithm provided in [39].

In sleep mode, the node does not attempt to measure nor send any data, and only consume power at a minimal rate. On the other hand, when in active mode, the node regularly measures the monitored environmental parameters, then sends the information to a station using short message service (SMS). All the measurement and transmission activities as well as the workload programmed in Active mode consume power. The principal parameters of the sensor node and the scenario are summarized in Table 1. These parameters are taken from an experimental node derived and implemented for research purposes.

In order to make the node useful, we want to maximize the number of measurement values collected. However, as the battery is only able to keep the node survive for a certain time, it needs to go in sleep mode for specific periods. To ensure the monitoring remains sufficiently continuous, we also do not want no measurements to be performed for too long a time. Finally, as the node needs to work in days with similar conditions, its final battery level of a day would not be lower than the initial one.

For this scenario, a time schedule for this sensor node needs to be determined. The fitness function is defined with four components:

(7) $$\Phi =\Phi_{1}+\Phi_{2}+\Phi_{3}+\Phi_{4},$$

in which $\Phi_{1}$ represents the term related to the number of measurements that we want to maximize, $\Phi_{2}$ is the term used to penalize the long time periods when there no measurement is taken, $\Phi_{3}$ is to penalize the schedule if the node is out of battery before the end of simulation, and $\Phi_{4}$ is to penalize the schedule if the final battery level is lower than the initial one. More specifically,

(8) $$\Phi_{1}=-k_{\eta }\eta ,$$

(9) $$\Phi_{2}=\sum_{i=1}^{\eta }k_{\tau 1}\Delta \tau_{i}+k_{\tau 2}\Delta \tau_{i}^{2},$$

(10) $$\Phi_{3}=k_{T1}\left(T-\tilde{T}\right)+k_{T2}{\left(T-\tilde{T}\right)}^{2},$$

(11) $$\Phi_{4}=\left\{\begin{array}{l} k_{L1}\left(L_{e}-L_{s}\right)+k_{L2}{\left(L_{e}-L_{s}\right)}^{2}\;\mathrm{if}\;L_{e}<L_{s},\\ 0\;\mathrm{if}\;L_{e}\geq L_{s}, \end{array}\right.$$

where η is the total number of performed measurements; $\Delta \tau_{i}$ is the time difference between two consecutive measurements; $\tilde{T}$ is the time moment when the battery runs out, or is equal to T if this does not happen; $L_{s}$ and $L_{e}$ are initial and final battery levels, respectively; and $k_{\eta }$, $k_{\tau 1}$, $k_{\tau 2}$, $k_{T1}$, $k_{T2}$, $k_{L1}$, $k_{L2}$ are constant weights. Note that quadratic functions are used in $\Phi_{2-4}$ to help the algorithm converges more quickly.

The values of principal parameters used in the VLC-GA are given in Table 2. Like in classical GAs, the population size is the number of individuals, i.e., the number of network schedule samples, per generation. A bigger population size means the algorithm explores more possibilities in each generation and would converges more quickly with respect to the number of generations, but the evaluation workload in one generation is proportionally higher. The rates to perform mutation and crossover operations are chosen as 80% in total so that a high number of new individuals are introduced in every generation, while the selection rate is kept not too small so that a sufficient number of elite individuals are preserved. When a mutation operation is performed on an individual, we want to introduce a few modifications so that only a small amount of the chromosome is changed for an individual. Otherwise, if too many modifications are taken, it would be hard for the algorithm to identify which ones of them cause good or bad effects on the individuals, and that would make the population quality very unstable over generations. In this case, a total rate of 10% is chosen for insertion, removal and shift operations, while the rate for copy operation is 90%. Finally, the weighting constants are chosen based on how much we want to penalize the corresponding terms, while also considering a balance of the magnitude between them.

**Figure 12:**
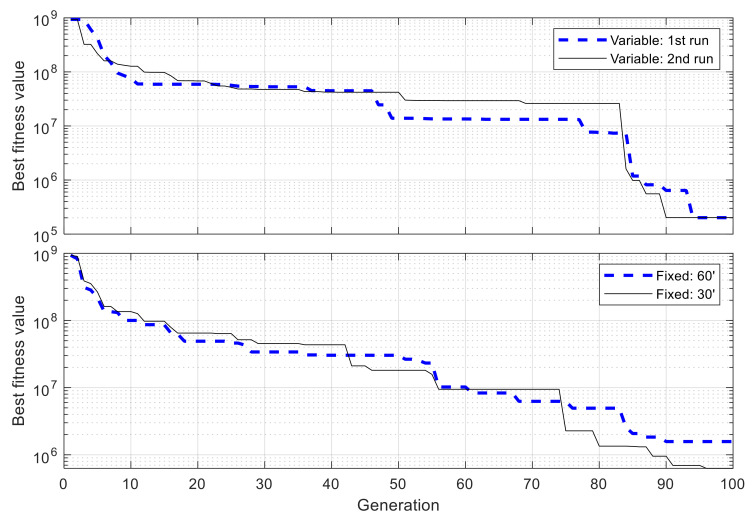
Progress of best fitness value using variable- and fixed-length chromosomes in single-node case.

**Figure 13:**
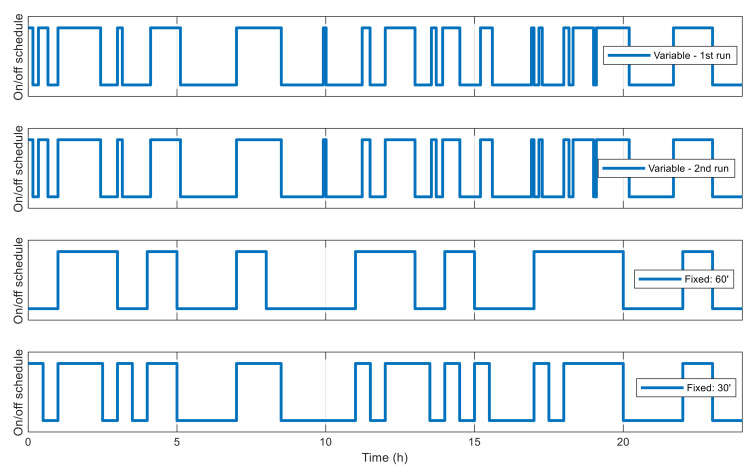
Best network schedules in single-node case.

With above configuration, the GA is executed two times. For a comparison of performance, two other GAs are used to optimize the same network, but using fixed-length chromosomes, i.e., the whole simulation time is divided into fixed and identical blocks of 60 min and 30 min in length, respectively, and only the state used in each time block needs to be determined by the algorithms, but not the block’s starting and ending times. From Figure 12 it can be observed that in both runs with VLC, the best fitness value converges consistently and efficiently with an exponential-like form. After 100 generations, the best fitness value is 2.010 × 10^5^ and 2.016 × 10^5^ for the first run and second run, respectively, which are very closed to each other, with 0.26% of difference, showing the reliability of the algorithm’s convergence performance and obtained results. For fixed-length cases with time blocks of 60 min and 30 min, the obtained fitness values are 1.574 × 10^6^ and 6.283 × 10^5^, respectively, due to the low adaptiveness of fixed time blocks. The best resulting schedules from these algorithms are shown in Figure 13, and those with VLC overlap exactly one over the other, with 18 active intervals per day, corresponding to 44% of the time in a day.

**Figure 14:**
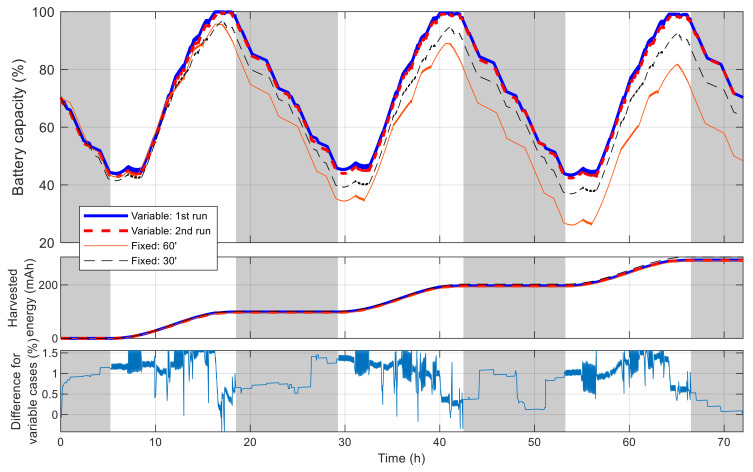
Battery capacity of best sensor node in single-node case.

**Figure 15:**
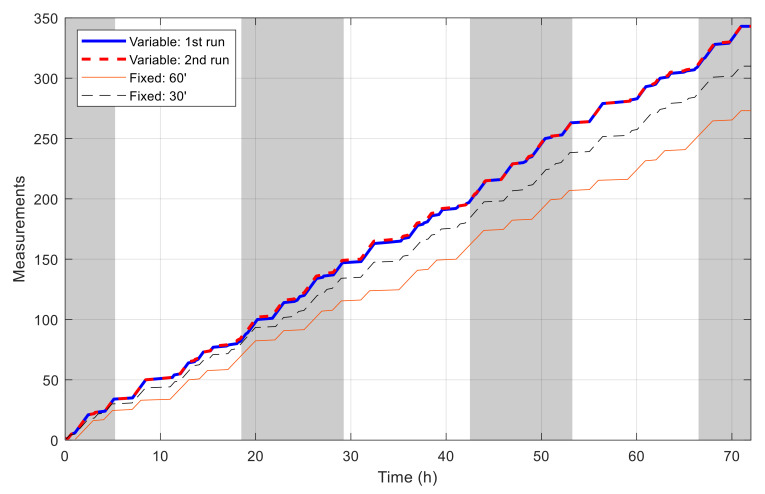
Number of measurements by time in single-node case.

Figure 14 shows the battery capacity percentage of the sensor node along with the cumulated harvested solar energy when simulated with the best schedules resulted from the aforementioned algorithms. Daytime and nighttime are also revealed in this figure by white and gray strips. With the initial and collected solar energy, the node is not able to cover all time in the day and the schedule is distributed to maximize the number of measurements. The difference of the capacity in the two simulations using the schedules resulted with VLC is minimal, with the maximal value of 1.56% and the root mean square error of 0.71%. In both simulations, the node performs 343 measurements, and the cumulated number of measurements grows similarly and gives a staircase-like form, as shown in Figure 15. The longest interval without measurement in the two runs is 1.962 and 1.957 h. Meanwhile, for simulations using the schedules resulted with fixed time blocks, it can be observed that the final battery level degrades gradually and is lower than the initial one by 21.8% and 6.4%, corresponding to the time blocks of 60 min and 30 min, respectively. These results show that, thanks to the flexibility of VLC, the resultant schedule clearly outperforms that obtained with fixed time blocks.

### 5.2. Multiple-Node Case

In this case, suppose the same environmental parameters are monitored, but more nodes are used together for this purpose. The scenario is extended to a network with three nodes labeled from 1 to 3. The configuration of these nodes is similar to that in the first case, except that their maximal battery capacity is now 3500 mAh, 5250 mAh (50% higher than that of node 1), and 7000 mAh (100% higher than that of node 1), respectively. All parameters of the VLC-GA remain unchanged. For a comparison of performance, a GA with fixed time blocks of 30 min is carried out.

**Figure 16:**
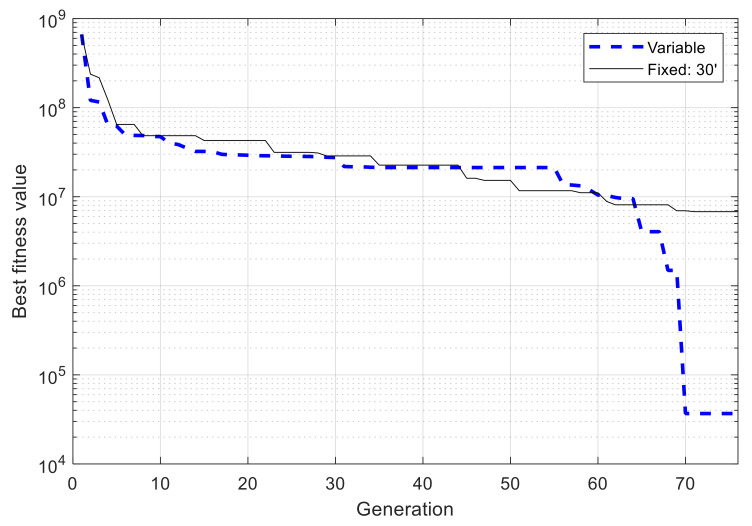
Progress of best fitness value using variable- and fixed-length chromosomes in multiple-node case.

**Figure 17:**
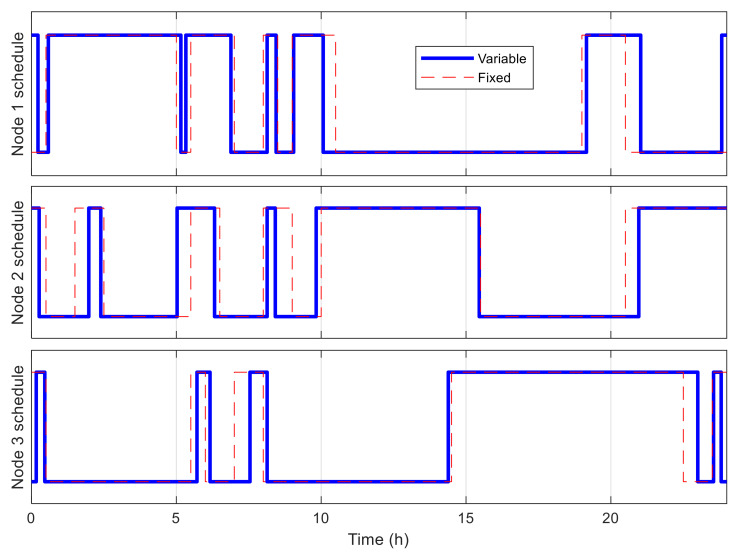
Best network schedule in multiple-node case.

**Figure 18:**
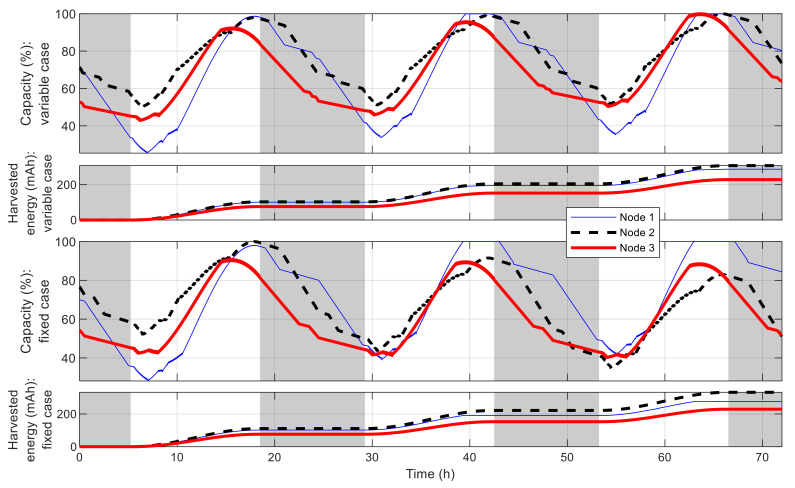
Battery capacity of best sensor node in multiple-node case.

**Figure 19:**
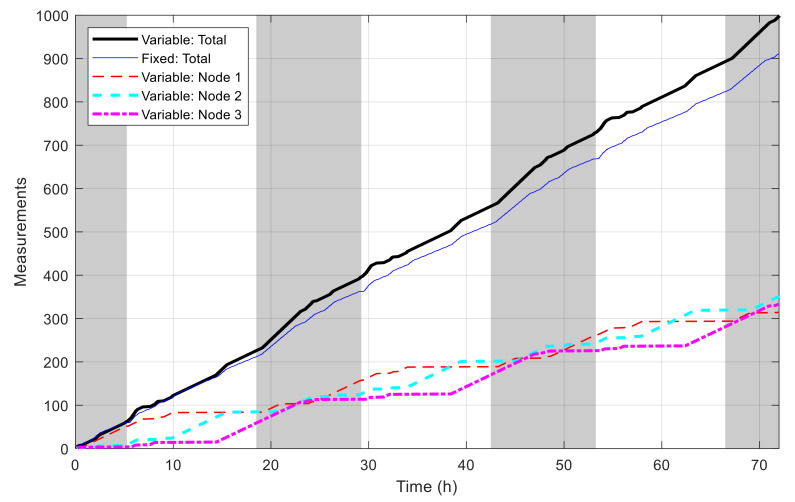
Number of measurements by time in multiple-node case.

Figure 16 shows the best fitness value, which decreases in an exponential-like manner to 3.680 × 10^4^ when using VLC, and to 8.129 × 10^4^ when using fixed time blocks. The obtained best network schedules in both cases are shown in Figure 17. Using VLC, the active time coverage in a day for the three nodes individually are 41%, 46%, and 43%, respectively, but their combination makes a coverage of 95% of time in a day. The battery capacity percentage of the nodes when simulated with this schedule is given in Figure 18, and none of them has a final level lower than the initial one, whereas for those of nodes 2 and 3 when using fixed time blocks, the final levels are 25.5% and 2.8% lower than the initial ones, respectively. The cumulated number of measurements is given in Figure 19, which is now smoother than that in the previous scenario. At the end of simulation, the nodes perform 315, 351 and 332 measurements individually, or 998 in total, whereas the total number of measurements when using fixed time blocks is 912, which is 8.6% lower. The results of this case also show that the resultant schedule obtained with VLC is better optimized than the one obtained with fixed time blocks.

## 6. Conclusions

Schedule optimization is critical for the deployment of sensor networks and is a non-trivial problem with large-scale and complex search space, such that it is not suitable to use deterministic approach, but stochastic and metaheuristic ones instead, including particle swarm optimization (PSO) [11], ant colony optimization (ACO) [10], bacterial foraging algorithm (BFA) [14], monarch butterfly optimization (MBO) [15], earthworm optimization algorithm (EWA) [16], elephant herding optimization (EHO) [17], moth search (MS) algorithm [18], slime mould algorithm (SMA) [19], Harris hawks optimization (HHO) [20], etc., besides GA, which is the most basic and well-known algorithm. The VLC-GA method introduced in this paper is a proposal to extend classical GAs to solve the problem when the schedule structure is variable. The test cases presented are rather simple, but they can clearly show that the proposed adaptation scheme is efficient and promising to address this problem, where the error curves are consistent between runs and have exponential form. Simulation results show that, thanks to the higher adaptiveness of VLC, the obtained network schedules are better optimized in comparison with those obtained when using fixed time blocks.

The technique introduced in this paper is an initial work, which presents several potential perspectives. First, it would also be possible to extend this work for application in sensor nodes with more than two states without difficulties, or even with dynamic schedules in which each node may change its state not only based on time, but also on other internal or external conditions. Furthermore, it might be interesting to adopt the same technique for other aforementioned evolutionary and swarm-intelligence optimization algorithms as well, so that they can work with a parameter array whose structure is changing. Another promising perspective is to combine the technique proposed in this paper with performance-improving methods, such as the information feedback models introduced in [23].

## Figures and Tables

**Table 1:** Principal simulation parameters in single-node case.

Parameter	Value
Number of nodes (*n*)	1
Maximal battery capacity	3500 mAh
Battery charging rate	0.8 W
Background power consumption rate in Sleep mode	0.05 W
Average power consumption rate in Active mode	0.17 W
Power consumption per measurement	0.22 Ws
Power consumption per SMS transmission	13.27 Ws
Installation location (latitude, longitude)	21.004°, 105.846°
Measurement rate in Active mode	5 min
Simulation time (*T*)	3 days

**Table 2:** Principal parameters of VLC-GA.

Parameter	Value
Population size	100
Selection rate	20%
Mutation rate	30%
Crossover rate	50%
Rates of mutation operations: Copy, Insertion, Removal, Shift	90%, 3.33%, 3.33%, 3.33%
$k_{\eta }$	1
$k_{\tau 1}$	1
$k_{\tau 2}$	10
$k_{T1}$	1 × 10^8^
$k_{T2}$	1 × 10^8^
$k_{L1}$	1 × 10^6^
$k_{L2}$	1 × 10^6^

## Data Availability

Not applicable.
